# Pest recognition based on multi-image feature localization and adaptive filtering fusion

**DOI:** 10.3389/fpls.2023.1282212

**Published:** 2023-11-17

**Authors:** Yanan Chen, Miao Chen, Minghui Guo, Jianji Wang, Nanning Zheng

**Affiliations:** ^1^ National Key Laboratory of Human-Machine Hybrid Augmented Intelligence, National Engineering Research Center for Visual Information and Applications, and Institute of Artificial Intelligence and Robotics, Xi’an Jiaotong University, Xi’an, China; ^2^ School of Software Engineering, Xi’an Jiaotong University, Xi’an, China

**Keywords:** pest recognition, multiple images, feature localization, feature filtering and fusion, smart agriculture

## Abstract

Accurate recognition of pest categories is crucial for effective pest control. Due to issues such as the large variation in pest appearance, low data quality, and complex real-world environments, pest recognition poses challenges in practical applications. At present, many models have made great efforts on the real scene dataset IP102, but the highest recognition accuracy is only 75%. To improve pest recognition in practice, this paper proposes a multi-image fusion recognition method. Considering that farmers have easy access to data, the method performs fusion recognition on multiple images of the same pest instead of the conventional single image. Specifically, the method first uses convolutional neural network (CNN) to extract feature maps from these images. Then, an effective feature localization module (EFLM) captures the feature maps outputted by all blocks of the last convolutional stage of the CNN, marks the regions with large activation values as pest locations, and then integrates and crops them to obtain the localized features. Next, the adaptive filtering fusion module (AFFM) learns gate masks and selection masks for these features to eliminate interference from useless information, and uses the attention mechanism to select beneficial features for fusion. Finally, the classifier categorizes the fused features and the soft voting (SV) module integrates these results to obtain the final pest category. The principle of the model is activation value localization, feature filtering and fusion, and voting integration. The experimental results indicate that the proposed method can train high-performance feature extractors and classifiers, achieving recognition accuracy of 73.9%, 99.8%, and 99.7% on IP102, D0, and ETP, respectively, surpassing most single models. The results also show that thanks to the positive role of each module, the accuracy of multi-image fusion recognition reaches the state-of-the-art level of 96.1%, 100%, and 100% on IP102, D0, and ETP using 5, 2, and 2 images, respectively, which meets the requirements of practical applications. Additionally, we have developed a web application that applies our research findings in practice to assist farmers in reliable pest identification and drive the advancement of smart agriculture.

## 1 Introduction

Pests pose one of the biggest threats to crop safety due to their extensive spread and rapid evolution. Effective pest control requires fast and accurate recognition of pest categories. Usually, agricultural practitioners identify pests by examining the surface of the crop, but which requires a high level of expertise and effort. With the help of computer vision technology, early researchers relied on carefully designed image processing programs to extract shallow features, and then trained machine learning classifiers for pest recognition. However, these models suffer from poor generalization and low accuracy (Liu and Wang, 2021). In recent years, deep learning technology has received extensive and profound research because of its simple modeling process, ability to extract deep features, and good recognition performance.

Although recent studies have achieved high pest recognition accuracy, most of them were conducted in laboratories with high-quality data, clear targets, few categories, and small datasets. However, the complex outdoor environment, different perspectives, and varying degrees of color and light changes will seriously affect their recognition performance in practice.

**Figure 1:**
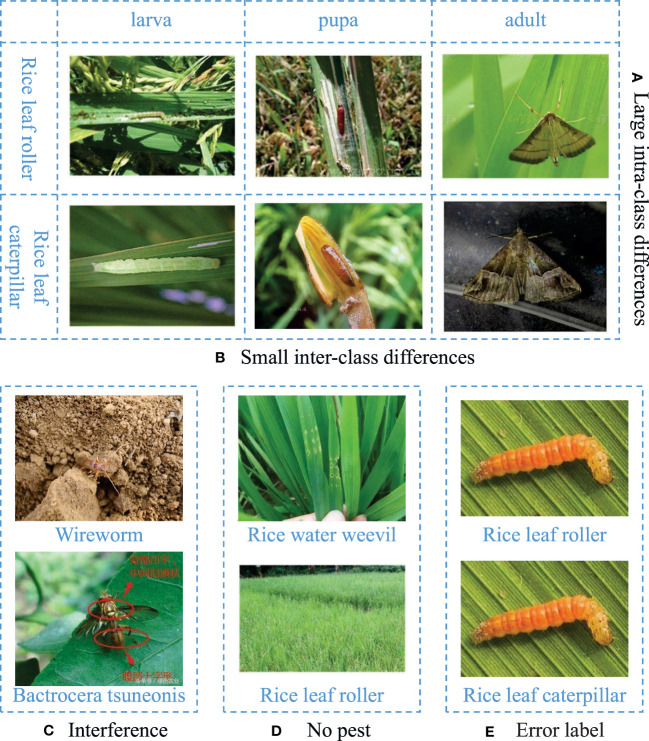
Issues with pests and data quality. **(A)** Large intra-class differences. From the horizontal view, rice leaf roller and rice leaf caterpillar have different appearances in larva, pupa and adult stages. **(B)** Small inter-class differences. From the vertical view, rice leaf roller and rice leaf caterpillar have similar appearances at the same stage. **(C)** Interference. **(D)** No pest. **(E)** Error label.

IP102 is the most widely studied large-scale pest dataset with images that match real-world scenarios (Wu et al., 2019). However, due to issues with the pests themselves and the quality of the IP102, pest recognition presents significant challenges in practical applications (Kong et al., 2022), as outlined below:

- Pests go through multiple growth stages throughout their lives, which have different external characteristics, leading to large intra-class differences, as shown in **Figure 1A**.
- Different pests may be similar at the same growth stage, which leads to small inter-class differences, as shown in **Figure 1B**.
- Some pests have similar appearances to their backgrounds, and some images from the Internet contain irrelevant elements, as shown in **Figure 1C**.
- Some images do not contain pests and only provide scenes of pest activity, as shown in **Figure 1D**.
- The images have various resolutions, and some are even blurry. Moreover, there are label errors in some images, as shown in **Figure 1E**.

Due to these practical issues, researchers have spent a lot of effort on recognition models based on a single image, but currently they can only achieve an accuracy of about 75% at best (Wang et al., 2023), with little improvement. It appears that the accuracy has reached a ceiling. However, in actual production, farmers may require close to 100% credibility to prevent incorrect pest control. This poses a great challenge for researchers. We consider the fact that pest infestation usually breaks out regionally and it is easy for farmers to obtain multiple images of the same pest category. To increase recognition credibility, we increase the amount of information input to the model and design information processing algorithms, thus proposing a fusion recognition method that integrates information from multiple images.

We propose the effective feature localization module (EFLM) and the adaptive filtering fusion module (AFFM) to improve pest recognition. To learn the accurate location of pests in complex background, in EFLM, we aggregate feature maps in the channel dimension to obtain the activation map, and then divide the feature location with a threshold that gradually increases to the average value of the activation map over the training epoch. The location information from multiple layers is lastly fused to construct the final location. To filter out incorrect and useless features, AFFM uses gate masks and selection masks to adaptively select optimal features from multiple images and then performs attention fusion. The experimental results show that the proposed method significantly improves the accuracy of identifying complex pest data and is suitable for practical scenarios. The main contributions of this paper are as follows:

1) The proposed EFLM helps the network quickly extract target features from complex backgrounds.
2) The proposed AFFM allows the network to automatically mine optimal features while suppressing incorrect and useless features, enhancing feature representation.
3) The proposed multi-image fusion recognition method significantly improves the accuracy of pest recognition, meeting practical application requirements.

## 2 Related research

### 2.1 Pest recognition based on CNN

Convolutional neural network (CNN) has shown outstanding performance as feature extractors in image recognition and have rapidly expanded to pest recognition applications. Ahmad Loti et al. (2021) compared traditional feature-based approaches with deep-learning feature-based approaches for extracting pest features from chili leaf images. The results proved that deep learning feature-based approaches performed better than traditional feature-based approaches. Cheng et al. (2017) used a deep residual convolutional neural network to recognize ten types of pests in 550 images. The study achieved an impressive accuracy of 98.67%, outperforming both support vector machine (SVM) and back propagation (BP) neural network significantly. In this paper, our research focus is not on the improvement of feature extractors, but on the localization and filtering fusion of extracted features. In fact, we can use any type of feature extractor, but CNN is the most commonly used.

### 2.2 Methods for improving pest recognition

To improve the recognition accuracy despite the limited size of pest datasets, a widely used and effective method is to apply transfer learning (Sharma et al., 2022). Transfer learning fine-tunes a CNN model that has been pre-trained on ImageNet (Deng et al., 2009) on a pest dataset to improve the model’s robustness. For example, Huang et al. (2022) first fine-tuned ResNet-50 to extract features and then used discriminant analysis to classify eight types of tomato pest images. The results demonstrated that transfer learning can reduce training time and improve recognition accuracy. Li et al. (2020) collected 5,629 images of ten common crop pests, used GrabCut and watershed algorithms to remove complex backgrounds, and then fine-tuned the GoogLeNet model to achieve a maximum accuracy of 98.91%.

In addition, researchers have proposed various methods to improve pest recognition accuracy from different perspectives. To enhance feature representation, Ullah et al. (2022) designed deeper CNN and achieved 100% accuracy on Deng pest dataset (Deng et al., 2018), surpassing SqueezeNet and GoogLeNet models. Liang et al. (2022) enhanced feature extraction by introducing depthwise separable convolution and squeeze-and-excitation (SE) module (Hu et al., 2018), achieving 93.66% accuracy on a dataset of 1,426 images containing nine rice pests and diseases. Wei et al. (2022) fused multi-scale features of images to achieve 98.2% recognition accuracy for 12 crop pests. To address imbalanced distribution of pest data, Yang et al. (2021) proposed a convolutional rebalancing network to extract more comprehensive features, achieving an accuracy of 97.58% on a dataset containing 18,391 images of rice diseases and pests. Furthermore, Hu et al. (2023) increased the number of samples using generative adversarial network (GAN), optimized the residual blocks using ConvNeXt in ResNet, and constructed a multi-scale dualbranch structure to extract features of different scales, achieving a recognition accuracy of 99.34% on a dataset containing four rice diseases and pests, surpassing classic CNN and transformer networks.

Although these methods have demonstrated impressive performance, it should be noted that their success is based on small datasets with high data quality. Therefore, it is difficult to maintain high performance in complex and constantly changing real-world environments.

### 2.3 Pest recognition on IP102

IP102 is a typical large-scale pest dataset with poor data quality, which is representative of real scene recognition. Based on transfer learning, five CNN models (VGG19, ResNet-50, EfficientNetB5, DenseNet-121, InceptionV3) were tested on IP102. The highest recognition accuracy achieved was only 71.98% for DenseNet-121 (Mohsin et al., 2022). Pest recognition on IP102 is challenging, but significant for smart agriculture. To take advantage of multi-model ensemble learning to improve recognition, Nanni et al. (2022) integrated six high-performance CNNs (EfficientNetB0, ResNet-50, GoogleNet, ShuffleNet, MobileNetV2, and DenseNet-201) and improved the Adam algorithm, obtaining an accuracy of 74.11%. Ensemble learning is effective but computationally demanding. As a general method, the key to improving performance still depends on the feature extraction ability of each model. Therefore, based on the new architecture vision transformer (ViT), Liu et al. (2022) proposed a latent semantic mask autoencoder to learn more discriminative feature representations, achieving the accuracy of 74.69%. Wang et al. (2023) further extended ViT to fine-grained recognition. They built an attention aggregating transformer with an information entropy selector to capture subtle differences between images, resulting in the highest accuracy of 75%. However, this method comes with a very high computational cost. By combining the above techniques, Xia et al. (2023) used multi-branch and multi-scale learning networks to extract fine-grained features and proposed a DNVT model combining DenseNet-201 and an improved ViT to enhance feature extraction. Finally, they achieved a maximum accuracy of 74.2% using ensemble learning. On the other hand, considering the main issues with IP102, Feng et al. (2022) designed a coarse-to-fine CNN to recursively filter complex backgrounds. They addressed occlusion problems by randomly deleting discrimination regions. Furthermore, they used a decoupling learning strategy to address class imbalance. Finally, their model recognition accuracy reached 74.61%.

Although pest recognition on IP102 has made great progress in the past few years, the state-of-the-art accuracy is only close to 75%. In this paper, we propose a multi-image fusion recognition method to improve the recognition accuracy of IP102.

## 3 Materials and methods

### 3.1 Datasets

**Figure 2:**
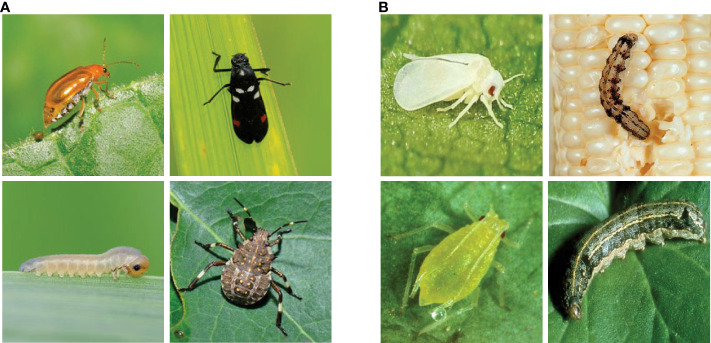
Image samples from D0 (Xie et al., 2018) and from ETP (Huang et al., 2022). **(A)** D0. **(B)** ETP.

The IP102 dataset contains a total of 75,222 images in 102 categories from 8 crops: rice, corn, wheat, beet, alfalfa, vitis, citrus, and mango (Wu et al., 2019) (see **Figure 1**). The training set has 45,095 images, the validation set has 7,508 images, and the test set has 22,169 images. To further demonstrate the effectiveness of our proposed method, we also conducted experiments on two high-quality small-scale pest datasets: D0 (Xie et al., 2018) and eight tomato pest (ETP) (Huang et al., 2022). Both datasets have higher image quality and only one growth stage for each category of pest, as illustrated in **Figure 2**. The D0 dataset contains 4,508 images of 40 common crop pest categories. The ETP dataset contains 609 original images of eight common tomato pests, which are increased to 4,263 images using image augmentation. Similar to Nanni et al. (2022), we used 70% of each class for training and the remaining 30% for testing.

### 3.2 Overview of the proposed method

**Figure 3:**
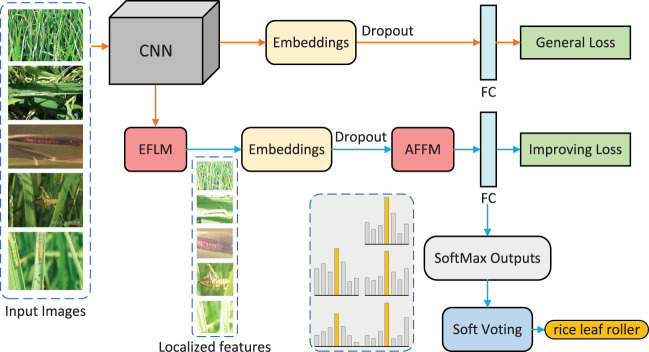
Overview of the proposed method.

The overall architecture of the multi-image fusion recognition method is shown in **Figure 3**, which consists of two branches: a general branch and an improving branch. The improving branch contains the EFLM and AFFM modules, which are used for feature localization and filtering fusion of multiple image features, respectively. The input of the model is multiple pest images of the same class.

The general branch uses a classic CNN as the backbone network to extract image features. Unlike conventional methods, the obtained feature embeddings are subjected to dropout processing to eliminate overfitting problems caused by excessive input information. The processed embeddings are then fed into the fully-connected (FC) layer, and the output results are optimized by the loss function to promote feature localization in the EFLM module.

In the improving branch, the EFLM module is used to locate fine features based on the activated feature maps in CNN. Then, the processed feature embeddings are sent to the AFFM module, where the features are filtered by gate masks and selection masks and then fused through multi-head self-attention network. Here, each image selects the most useful image-level features. Then, the fused embeddings are sent to the same FC layer as the general branch for classification. To obtain more accurate result, a soft voting strategy is adopted to integrate all outputs at the end.

### 3.3 Effective feature localization module

**Figure 4:**
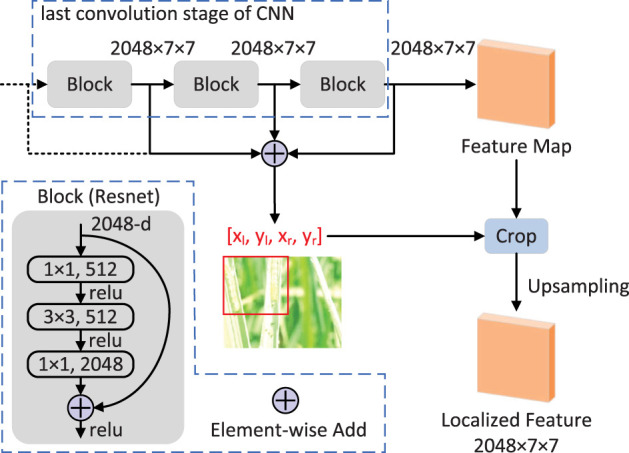
EFLM module.


The proposed EFLM module is shown in **Figure 4**, which is inspired by SCDA (Wei et al., 2017). To use deep features, we focus on all blocks in the last convolutional stage, whose output feature maps have the same height *h* and width *w*. The activated region of the feature map on the channel can indicate the semantically meaningful part of the target or some background noise. To eliminate noise and obtain accurate feature location, we first aggregate the feature maps in the channel dimension to obtain the activation map **A** ∈ ℝ^*h*xw^.

(1) $$A=\sum_{n=1}^{c}F_{n}$$

where *c* represents the number of channels in the feature maps, and ***F**_n_* represents the feature map of the *n*-th channel.

Then, we calculate the overall average value *Ā* of all positions in the activation map as a threshold to divide the regions with higher activation values, where the target is more likely to be located.


(2) $$\bar{A}=\frac{\sum_{i=1}^{w}\sum_{j=1}^{h}A_{\left(i,j\right)}}{w\times h}$$

Next, we consider that the model is more easily disturbed during the early stages of training and may not activate the correct feature locations. Therefore, we multiply the threshold by a parameter *λ* that linearly increases from 0 to 1 with the training epoch to gradually discover the feature locations. Specifically, the feature mask map ***M*** used for partitioning is represented as follows:


(3) $$M_{\left(i,j\right)}=\{\begin{array}{ll} 1 & \text{if}A_{\left(i,j\right)}>\text{λ}\bar{A}\\ 0 & \text{otherwise} \end{array}$$

During the training and validation phases, $\text{λ}=\frac{Epoch}{Epochs-1}\in \left[0,1\right]$ , where *Epoch* represents the current training epoch and *Epochs* represents the total number of training epochs. In the testing phase, λ takes the median value of 0.5. We have conducted multiple experiments and found that this setting can achieve the best results. In the mask map, ***M***_(*i, j*)_ = 1 represents that the channel at position (*i, j*) should be retained, while ***M***_(*i, j*)_ = 0 represents that the channel at position (*i, j*) should be removed.

Inspired by multi-layer integration to improve performance (Wei et al., 2017), we calculate the feature mask maps for all the block outputs of the last convolutional stage by equations (1), (2), (3). Taking ResNet-50 as an example, we aggregate the feature mask maps **M_conv_5a_**, **M_conv_5b_** *and* **M_conv_5c_**, and retain their intersection to obtain an aggregated mask map **M_f_** as follows:


(4) $${M_{f}}_{\left(i,j\right)}=\{\begin{array}{ll} 1 & \text{if}{M_{conv\_5a}}_{\left(i,j\right)}+{M_{conv\_5b}}_{\left(i,j\right)}+{M_{conv\_5c}}_{\left(i,j\right)}=3\\ 0 & \text{ otherwise} \end{array}$$

Then, we take the bounding box that contains the area of ${M_{f}}_{\left(i,j\right)}=1$ as the final determined feature location. If all positions in ***M_f_*** are 0, the bounding box is the box of the whole feature map. Finally, we use the bounding box to crop the feature maps and upsample them to the original size to obtain the localized features.

### 3.4 Adaptive filtering fusion module

**Figure 5:**
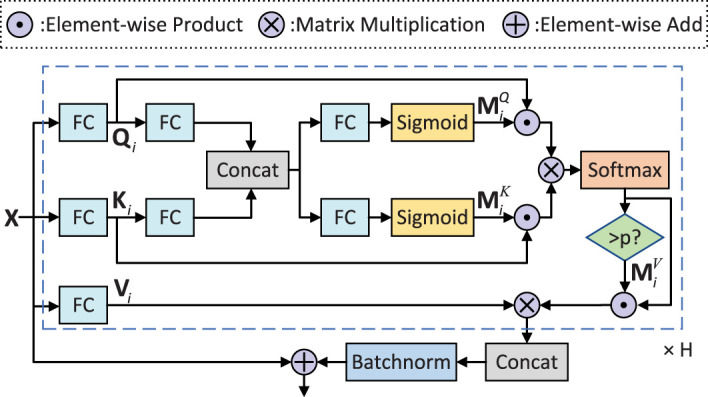
AFFM module.


The proposed AFFM is shown in **Figure 5**. The input of the module is the feature embedding matrix **X** ∈ ℝ*^n^* ^×^ *^d^* obtained by processing the multiple localized features, where *n* is the number of input images and *d* is the dimension of the embedding. The main framework of the module is a multi-head self-attention network. For the *i*-th head, the query matrix ***Q_i_***, key matrix ***K_i_***, and value matrix ***V_i_*** are obtained by linear transformation, respectively:

(5) $$\{\begin{array}{c} Q_{i}=XW_{i}^{Q}\\ K_{i}=XW_{i}^{K}\\ V_{i}=XW_{i}^{V} \end{array}$$

where $W_{i}^{Q}\in {\mathbb{R}}^{d\times \left(d_{k}/h\right)},W_{i}^{K}\in {\mathbb{R}}^{d\times \left(d_{k}/h\right)}$ and $W_{i}^{V}\in {\mathbb{R}}^{d\times \left(d_{v}/h\right)}$ represent the query parameter matrix, key parameter matrix, and value parameter matrix of the *i*-th head, respectively. *h* is the number of heads. *d_k_* and *d_v_* are the dimensions of them, both of which are set to *d* in this paper.

Considering the noisy background in pest images, we introduce a gating mechanism to filter out useless information. Specifically, we use the non-linear mapping function of the sigmoid function to adjust the information transmission of the query matrix and key matrix. We first linearly map the query matrix ***Q_i_*** and key matrix ***K_i_*** of the *i*-th head to a joint space and concatenate them to obtain a fusion matrix $G_{i}\in {\mathbb{R}}^{n\times 2\left(d/h\right)}$ :


(6) $$G_{i}=Concat\left(Q_{i}W_{G}^{Q}+b_{G}^{Q},\;K_{i}W_{G}^{K}+b_{G}^{K}\right)$$

where $W_{G}^{Q}\in {\mathbb{R}}^{\left(d/h\right)\times \left(d/h\right)}$ and $W_{G}^{K}\in {\mathbb{R}}^{\left(d/h\right)\times \left(d/h\right)}$ are learnable mapping matrices, and $b_{G}^{Q}\in {\mathbb{R}}^{1\times \left(d/h\right)}$ and $b_{G}^{K}\in {\mathbb{R}}^{1\times \left(d/h\right)}$ are bias vectors. Next, we apply two FC layers followed by sigmoid function to obtain the query mask $M_{i}^{Q}\in {\mathbb{R}}^{n\times \left(d/h\right)}$ and key mask $M_{i}^{K}\in {\mathbb{R}}^{n\times \left(d/h\right)}$ , respectively:


(7) $$\{\begin{array}{l} M_{i}^{Q}=\sigma \left(G_{i}W_{M}^{Q}+b_{M}^{Q}\right)\\ M_{i}^{K}=\sigma \left(G_{i}W_{M}^{K}+b_{M}^{K}\right) \end{array}$$

where *σ*(·) is sigmoid function, and $W_{M}^{Q}\in {\mathbb{R}}^{2\left(d/h\right)\times \left(d/h\right)},W_{\text{M}}^{K}\in {\mathbb{R}}^{2\left(d/h\right)\times \left(d/h\right)},\;b_{M}^{Q}\in {\mathbb{R}}^{1\times \left(d/h\right)}$ and $b_{M}^{K}\in {\mathbb{R}}^{1\times \left(d/h\right)}$ are the learnable FC layer parameters.

To further remove image interference such as label errors and no features, we designed a selection mask to force discard image embeddings with attention scores smaller than probability *p*, which can be represented as a value mask $M_{i}^{V}\in {\mathbb{R}}^{n\times n}$ :


(8) $$M_{i}^{V}=\{\begin{array}{ll} 1 & \text{otherwise}\\ 0 & \text{if }\alpha <p \end{array}$$

In summary, in the calculation of self-attention within each head, the *i*-th head output ***h_i_*** ∈ R*^n^* ^×(^ *^d/h^* ^)^ is represented as follows:


(9) $$h_{i}=M_{i}^{V}⊙Softmax\left(\frac{\left(M_{i}^{Q}⊙Q_{i}\right){\left(M_{i}^{K}⊙K_{i}\right)}^{\text{T}}}{\sqrt{d_{k}}}\right)V_{i}$$

Finally, after aggregating all the heads and applying batch normalization (BN), we obtain the enhanced feature embeddings, which can be represented as:


(10) $$\;F_{AFFM}\left(X\right)=BN\left(Concat\left(h_{1},\ldots ,h_{h}\right)\right)+X$$

The AFFM module enhances the feature selection ability of attention, resulting in more discriminative features. Specifically, the gate mask and selection mask suppress the transmission of irrelevant information in the image, improving the quality of the features. The multi-head self-attention network further filters and fuses the features to extract the most beneficial features for recognizing each image. This process can be adaptively adjusted through learnable parameters.

### 3.5 Soft voting

To achieve more accurate recognition by merging multiple results, the soft voting (SV) strategy is used in practical applications to determine a category for these images. Specifically, given the feature embedding matrix ***X*** ∈ ℝ*^n^* ^×^ *^d^* after fusion of *n* images, a FC layer and a softmax layer are used to obtain the probability matrix ***p*** ∈ ℝ*^n^* ^×^ *^m^*, where *m* is the total number of categories. SV calculates the average of the predicted probabilities for *n* images:


(11) $$P_{j}=\frac{\sum_{i=1}^{n}p_{ij}}{n},j\in \left[1,m\right]$$

where ***P_j_*** denotes the probability of being predicted as category *j* after soft voting. SV takes the category with the highest probability as the final result.

### 3.6 Loss function

The general branch and the improving branch share a FC layer, and both use the predicted category and true category to calculate the cross-entropy loss:


(12) $$L_{general}=-\log\;P_{ɡ}\left(t\right)$$

(13) $$L_{improving}=-\log\;P_{i}\left(t\right)$$

where *t* is the ground truth label of the input image, *P_ɡ_* and *P_i_* represent the classification probabilities output by the general branch and the improving branch, respectively. The two losses are jointly optimized during training and mutually promote each other. The overall loss is defined as the sum of them:


(14) $$L_{total}=L_{general}+L_{improving}$$

## 4 Experiments and discussion

### 4.1 Experimental settings

We use ResNet-50 as the backbone network and load the parameters pre-trained on ImageNet. Stochastic gradient descent (SGD) with a momentum of 0.9 and weight decay of 1e-4 is used as the optimizer. We set the batch size to 16, and each image in the batch selects 4 more images from its own category as positive samples, i.e., the number *N_t_* of training samples per group is 5. The initial learning rate is set to 0.001 and is decayed by 0.1 after 10 epochs, under the condition of a total of 20 epochs. All experiments are conducted on an RTX 3090 GPU. The threshold probability *p* for selecting masks is set to $\frac{1}{2N_{t}}$ .

We use Accuracy and weighted F1-score as evaluation metrics, which are denoted as *Acc* and *F*1, respectively. *Acc* calculates the proportion of correctly classified images to the total number of images, while *F*1 combines Precision (*P*) and Recall (*R*) to prevent individual classes from affecting *Acc* and causing poor evaluation when the data is imbalanced. A higher *F*1 indicates a more robust model. The formulas for calculating these metrics are as follows:


(15) $$Acc=\frac{TP+TN}{TP+TN+FN+FP}$$

(16) $$P=\frac{TP}{TP+FP}$$

(17) $$R=\frac{TP}{TP+FN}$$

(18) $$F1=\frac{2PR}{P+R}$$

where *TP*, *FP*, *TN*, and *FN* stand for true positive, false positive, true negative, and false negative, respectively.

### 4.2 Performance comparison

We conducted experiments on three pest datasets and compared our results with the current state-of-the-art methods. For methods that were not tested on the corresponding dataset, we reproduced them based on the best configuration in our environment and marked them with an *. Methods without a mark use the results from the original paper. To maintain objectivity, we listed the backbone of all models.

#### 4.2.1 Comparison on IP102

We compared our method with the 12 latest methods, as shown in **Table 1**. Our general branch achieved 73.9% accuracy with only trained CNN and FC layers, outperforming most CNN-based methods. This is because we trained a robust feature extractor and classifier by selecting optimal features from low-quality data. Pre-trained CNN based on transfer learning is the basis of many methods, including ours, and the reason for their low performance may be mainly due to the lack of information interaction. MMAL (Zhang et al., 2021) is a fine-grained recognition method that has some improvement compared to the baseline, but its performance is limited due to small pests and low image resolution. BCL (Zhu et al., 2022) uses an advanced supervised contrastive learning strategy to balance samples, but it is limited by the number of samples in each batch, and the feature extraction performance is relatively weak. Although PCNet (Zheng et al., 2023) is a lightweight framework, it also adds a complex coordinate attention mechanism and feature fusion process, and its accuracy is not as good as our trained general CNN. Among CNN-based methods, only MS-ALN+DL (Feng et al., 2022) exceeded our general branch. Although it solves the problems of localization, occlusion, and class imbalance, the model is extremely complex and the obtained accuracy is still not enough for application.

**Table 1:** Performance comparison on IP102 dataset.

Methods	Backbone	Acc	F1
Pre-trained ResNet-50* (He et al., 2016)	ResNet-50	72.0	71.5
Pre-trained DenseNet-169* (Huang et al., 2017)	DenseNet-169	72.8	72.5
MMAL* (Zhang et al., 2021)	ResNet-50	73.1	72.8
BCL* (Zhu et al., 2022)	ResNet-50	73.1	72.8
ViT (Dosovitskiy et al., 2020)	Transformer	73.4	72.7
PCNet (Zheng et al., 2023)	EfficientNetV2	73.7	–
**Ours (general branch)**	**ResNet-50**	**73.9**	**73.6**
CNNs Ensemble + Exp + ExpLR (Nanni et al., 2022)	EfficientNetB0 + ResNet-50 + GoogleNet + ShuffleNet + MobileNetV2 + DenseNet-201	74.1	73.0
MMALNet + DNVT + ResNet-50 + Ensemble (Xia et al., 2023)	ResNet-50 + DenseNet-201 + Transformer	74.2	67.8
MS-ALN + DL (Feng et al., 2022)	ResNet-50	74.6	67.8
FRCF + LSMAE (Liu et al., 2022)	Transformer	74.7	74.4
AA-Trans (Wang et al., 2023)	Transformer	75.0	–
**Ours (improving branch)**	**ResNet-50**	**96.1**	**95.9**

The bolded lines are the results obtained by our method, to emphasize.

Compared with transformer-based and ensemble-based methods, most of them exceeded the accuracy of our general branch. This is mainly because they use more advanced and complex feature extractors and other special processing techniques to improve feature representation ability, which also makes their models complex and computationally expensive.

The biggest feature of our method lies in its practical applications, not just in performance comparison. The improving branch can achieve recognition results far beyond other complex models. This is primarily achieved by leveraging a trained efficient CNN, multiple image information, feature localization, filtering fusion, and voting. For example, when inputting 5 images to be recognized at the same time, our method achieved 96.1% accuracy. Although additional information is introduced, considering the significant improvement in accuracy and the low cost of image acquisition in practical applications, our method has great practical significance.

#### 4.2.2 Comparison on D0 and ETP

Our method not only has excellent performance on low-quality dataset IP102 but also has a positive effect on normal datasets. **Tables 2** and **3** respectively show the comparison results of the D0 dataset and ETP dataset with other state-of-the-art methods. Due to the high image quality, all models achieved high accuracy on the two datasets. Our method trained a high-performance feature extractor and classifier through strategic fusion of multiple information. As a result, on the general branch, using the same feature extractor and classifier, our method outperformed the pre-trained CNN in terms of performance. However, the general branch’s performance was inferior to that of the model ensemble-based methods, primarily because they leverage the advantages of multiple feature extractors to obtain better feature representations. However, this approach requires significant computational resources and storage space. MMAL (Zhang et al., 2021) performs well in classifying images of pests with multiple stages. However, its improvement in recognition is not significant for dataset D0, which contains only single-stage images. For ACEDSNET (Li et al., 2021), a lightweight model constructed by researchers, its low accuracy may be due to its inability to effectively extract important features. The performance of ResNet-50 + DA (Huang et al., 2022) is weaker than our experiment’s pre-trained ResNet-50. We discovered that a possible reason is that they used a smaller learning rate, resulting in insufficient training of the network. It is worth mentioning that on these two datasets, using the improving branch with only two input images can achieve almost 100% accuracy, surpassing other methods, and its simplicity and efficiency make it suitable for practical applications.

**Table 2:** Performance comparison on D0 dataset.

Methods	Backbone	Acc	F1
Pre-trained ResNet-50* (He et al., 2016)	ResNet-50	99.4	99.4
Pre-trained DenseNet-169* (Huang et al., 2017)	DenseNet-169	99.6	99.6
MMAL* (Zhang et al., 2021)	ResNet-50	99.8	99.8
**Ours (general branch)**	**ResNet-50**	**99.8**	**99.8**
CNNs Ensemble + Exp + ExpLR (Nanni et al., 2022)	EfficientNetB0 + ResNet-50 + GoogleNet + ShuffleNet + MobileNetV2 + DenseNet-201	99.8	99.7
MMALNet + DNVT + ResNet-50 + Ensemble (Xia et al., 2023)	ResNet-50 + DenseNet-201 + Transformer	99.9	99.9
**Ours (improving branch)**	**ResNet-50**	**100**	**100**

The bolded lines are the results obtained by our method, to emphasize.

**Table 3:** Performance comparison on ETP dataset.

Methods	Backbone	Acc	F1
ACEDSNet (Li et al., 2021)	MobileNet	96.8	–
ResNet-50 + DA (Huang et al., 2022)	ResNet-50	97.1	96.1
Pre-trained DenseNet-169* (Huang et al., 2017)	DenseNet-169	99.2	99.2
Pre-trained ResNet-50* (He et al., 2016)	ResNet-50	99.7	99.7
**Ours (general branch)**	**ResNet-50**	**99.7**	**99.7**
**Ours (improving branch)**	**ResNet-50**	**100**	**100**

The bolded lines are the results obtained by our method, to emphasize.

### 4.3 Ablation study

We conducted ablation studies on IP102 to analyze the impact of the methods used in our model on classification. Each group is trained with 5 positive samples during training. During testing, the general branch uses single image, while the improving branch uses 5 images of the same category per group by default to improve recognition.

#### 4.3.1 Module ablation

To verify the effectiveness of our proposed framework, we conducted ablation studies on the main modules, and the results are shown in **Table 4**.

**Table 4:** Module ablation.

No.	Branch	Method	Acc
1	Improving branch	Full	96.1
2		w/o EFLM	95.0
3		w/o AFFM	90.9
		w/o SV	94.2
5	General branch	Trained ResNet-50	73.9
6	No	Pre-tained ResNet-50	72.0
7		w/i Multiple images & Hard voting	88.7

In the improving branch, we use the full module as the baseline, and remove each major module separately for training and testing, as shown in No.1-4. As a comparison, we also listed the test results of the fully trained network in the general branch, as shown in No.5. Comparing No.1 and No.5, the improving branch can achieve recognition accuracy far higher than the general branch, mainly because the improving branch can merge multiple image information and eliminate the adverse effects of bad information during separate image classification. Comparing No.1 and No.2, it can be found that the accuracy decreases by 1.1% without the EFLM module, indicating that EFLM is useful as it can locate features and remove some noisy information. Comparing No.1 and No.3, it can be found that the accuracy decreases the most by 5.2% without the AFFM module, which indicates that the filtering and fusion of information has a great impact on the model, as it can filter out useless information adaptively and fuse useful information to achieve more accurate classification. Comparing No.1 and No.4, it can be found that the soft voting method can increase the recognition accuracy by 1.9%. As the last step in determining recognition results, it can avoid misclassifying images without obvious features.

To ablate multiple image information, we fine-tuned the model on a pre-trained ResNet-50 and applied a hard voting strategy to the same number of image classification results, as shown in No.6-7. Hard voting takes the most majority of the voted categories as the final result, in line with human subjective consciousness. Comparing No.1 and No.7, it can be found that our multi-image fusion recognition method can achieve higher accuracy. Furthermore, comparing No.6 and No.7 also shows that introducing multiple image information can bring simpler and more practical accuracy improvements.

#### 4.3.2 The impact of the number of training positive samples and the number of test images

**Figure 6:**
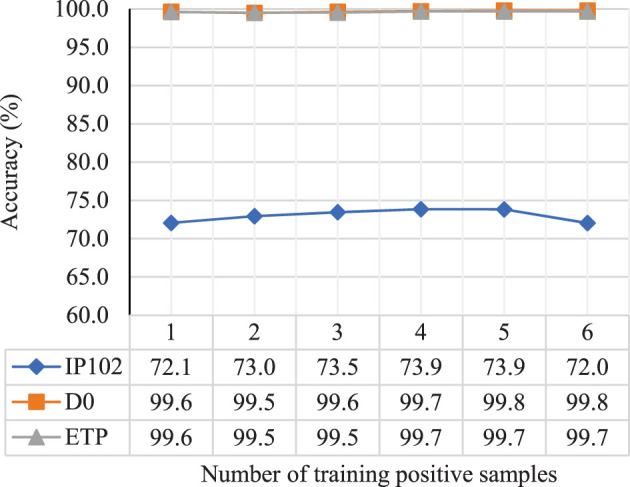
The impact of the number of training samples in each group on the performance of CNN and FC after training.

The number of training samples in each group mainly determines the quality of CNN and FC after training. We trained the model with the number of positive samples ranging from 1 to 6 and tested it with a single image input to the general branch, drawing a curve graph as shown in **Figure 6**.

According to the results, when the number of training images is 1, the AFFM does not work due to the lack of image interaction, resulting in lower performance of the trained model. When the number of images is greater than 1, the performance of the trained model improves as the number of images increases, reaching its maximum value when the number of images is 4 or 5. However, the performance no longer increases or even decreases thereafter. This could be due to the fact that when there are too many training images, a few low-quality images are filtered out, and more normal images tend to participate equally in the fusion process, leading to a decrease in the optimal feature selection effect.

**Figure 7:**
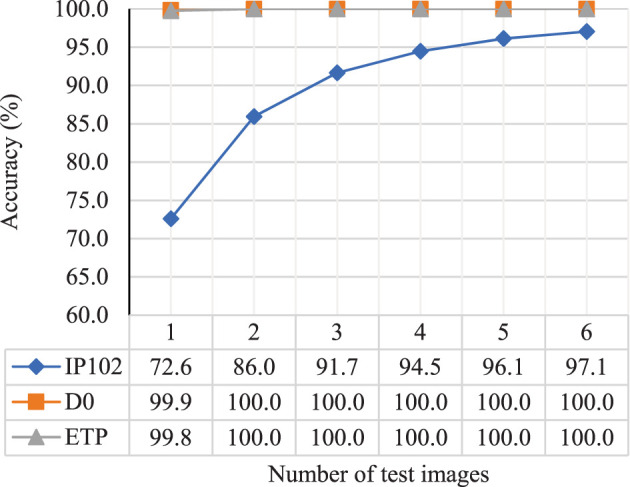
The impact of the number of test images on the accuracy of the improving branch.

The number of additional images introduced during testing determines the recognition performance of the improving branch in practical applications. Therefore, we used the network trained with 5 positive samples as the basis and conducted experiments by changing the number of test images from 1 to 6, as shown in **Figure 7**.

From **Figure 7**, it can be seen that the accuracy of the improving branch increases as the number of test images increases, demonstrating the effectiveness of EFLM, AFFM, and SV in extracting and fusing multiple image information. Specifically, for the high-quality datasets D0 and ETP, the accuracy reached 99.9% and 99.8%, respectively, when tested with a single image. When the number of test images increased by one more, the accuracy remained at the peak of 100%. However, for the low-quality dataset IP102, the increase in accuracy gradually slowed down as the number of test images increased. The accuracy reached 96.1% when there were 5 test images, and the increase in accuracy became minimal thereafter, indicating that the role of multiple image information was no longer significant.

#### 4.3.3 The impact of integrated localization and gradual localization

To test the impact of the number of integrated localization layers on accuracy, we used ResNet-50 as an example to conduct experiments on the last one block output, the integration of the last two block outputs, and the integration of all three block outputs of its last stage. All other modules were configured as “Full.” The results are presented in **Table 5**.

**Table 5:** The impact of integrated localization on accuracy.

Methods	Acc
Last one block output	95.8
Integration of the last two block outputs	95.9
Integration of all three block outputs	96.1

As shown in **Table 5**, the accuracy gradually increases as the number of integrated localization layers increases, demonstrating the effectiveness of multi-layer integrated localization. The small improvement in accuracy is mainly due to the fact that the fusion of information from 5 images enhanced recognition. We believe that integrated localization will play a greater role in fusing information from fewer images and in more complex image recognition tasks, relative to the small computational cost.

In the EFLM module, we linearly increase the activation map average value from 0 to the original value as the training progresses to gradually discover the feature location. The experiments show that without gradual localization, we achieved an accuracy of 95.7%, which is lower than 96.1% with gradual localization, demonstrating the effectiveness of gradual localization.

#### 4.3.4 The impact of information filtering

To test the impact of the information filtering in the AFFM module on accuracy, we conducted four separate experiments by combining the query and key masks as one set and the value mask as another set. The results are shown in **Table 6**.

**Table 6:** The impact of information filtering on accuracy.

No	Query & Key Mask	Value Mask	Acc
1	✘	✘	95.5
2	✔	✘	95.9
3	✘	✔	95.4
4	✔	✔	96.1

Although using 5 images in the improving branch helped to enhance recognition and narrow the accuracy gap, we can also draw the following conclusions from the table. Firstly, the query and key masks played a crucial role in information filtering. Secondly, filtering information with a separate value mask had a negative effect on the results, but when combined with the query and key masks, the best experimental results were obtained. This is mainly because the value mask is a forced filtering method, which removes features that the query and key masks consider to be useless. However, when there are no query and key masks, direct forced filtering can cause useful information to be erroneously removed. In summary, due to the background of the pest itself and the issues of the dataset, the images contain significant interference, making adaptive information filtering necessary.

### 4.4 Visualization

**Figure 8:**
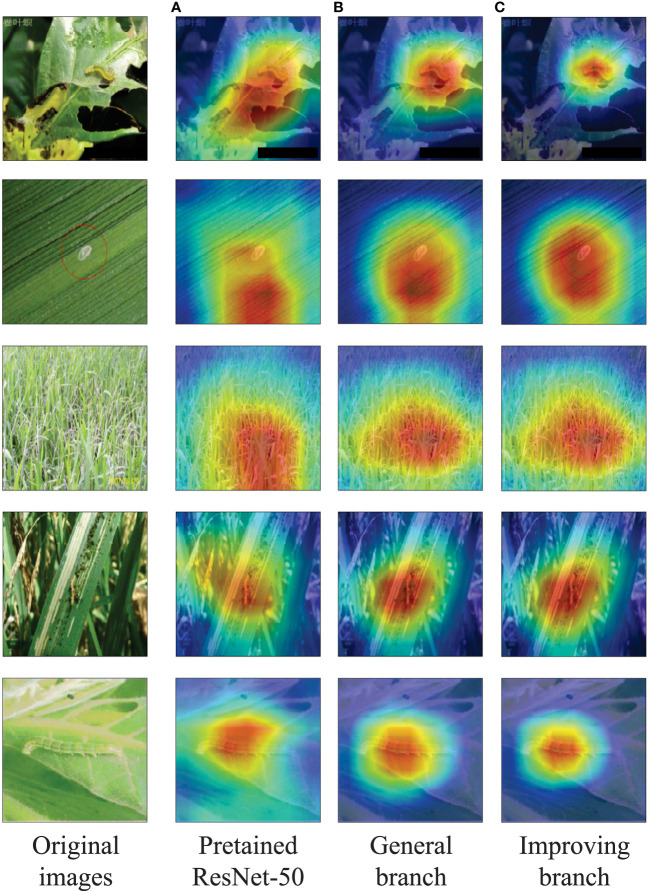
Visualization results. **(A)** Pretained ResNet-50. **(B)** General branch. **(C)** Improving branch.

Grad-CAM (Selvaraju et al., 2017) is a feature visualization method that uses gradients to compute the attention regions of the network in the feature maps. We applied Grad-CAM to compare pre-trained ResNet-50 with our general branch and improving branch, both trained with 5 positive samples. **Figure 8** shows the visualization results, where the labels of the original images are all rice leaf rollers.

The results show that the pre-trained ResNet-50 network can only roughly focus on the target and even incorrectly locate it. However, with the enhancement of multiple positive samples, our model has better discriminative ability, and the general branch can accurately focus on the target. The attention performance of the improving branch is affected by the quality and quantity of the test images. With the help of additional information, the improving branch can more accurately focus on the target, which to some extent promotes subsequent feature localization and fusion, thereby achieving more accurate recognition accuracy.

### 4.5 Application deployment

**Figure 9:**
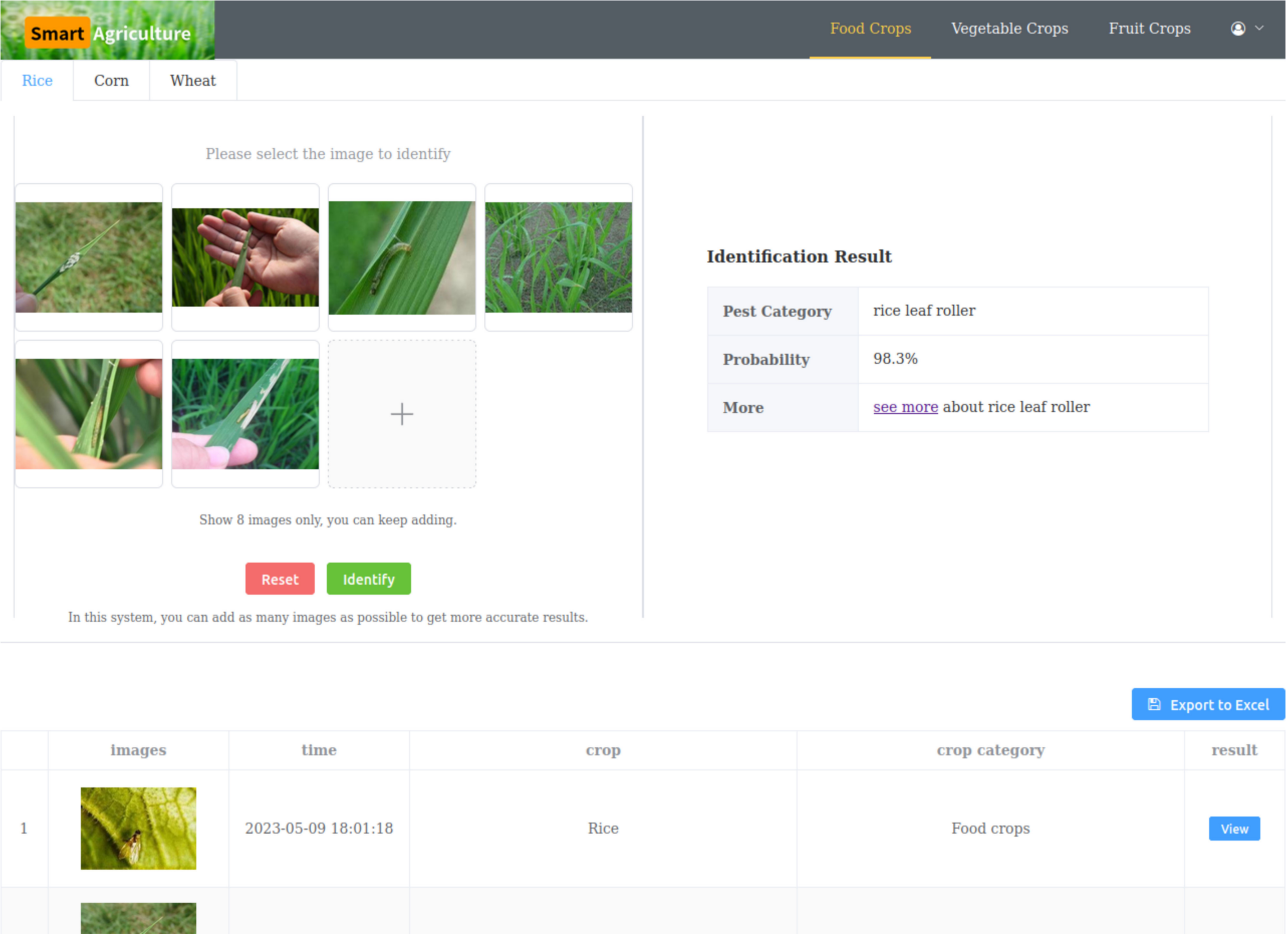
Web application.

To apply our research results practically, we built a web application called “Smart Agriculture,” which can run on personal computers and mobile phones, as shown in **Figure 9**. The application includes multiple common crops for users to choose from. On the left side of the page, users can upload pest images for identification without restrictions to improve identification credibility. On the right side of the page, the identification results are displayed, including the pest type, probability, and a link to learn more about the identified pest. Below the page, the identification history can be displayed and exported to Excel for users to review repeatedly.

We deployed the model trained with 5 positive samples on a cloud server to receive requests from the web application. Regardless of how many images need to be identified, the model can give very high identification credibility. As shown in **Figure 7**, the more images users input, the higher accuracy they will get. The time taken by the model is related to the number of input images and the configuration of the cloud server. In general, results will be displayed within two seconds. However, when it comes to achieving highly accurate recognition results, the time spent is negligible.

### 4.6 Discussion

We achieved very high recognition accuracy by the improving branch, but the main reason for the improvement is the introduction of additional images. Our method only makes the most of this information as much as possible. Comparing No.6 and No.7 in **Table 4**, it can be observed that with the input of 5 images, the basic hard voting fusion recognition method can achieve an accuracy of 88.7%, which is a significant improvement of 16.7% compared to 72.0% for single image recognition. Through the fusion process of EFLM, AFFM, and SV, we achieved an accuracy of 96.1% in the improving branch, which is only 7.4% higher than the basic hard voting method. This may be the true improvement ability of our proposed method. Additionally, the input requirement of multiple images also brings inconvenience to pest recognition. On the one hand, during model training, the dataset is required to have as rich pest images as possible to increase the feature richness of input images, thereby improving the fusion ability of the network. On the other hand, according to **Figure 7**, reliable recognition results can only be obtained by inputting as many images as possible in practical applications. Although farmers can easily capture photos, this increases their inconvenience and the computational consumption.

Therefore, looking ahead to the future, we will optimize the fusion recognition method to achieve high recognition accuracy with fewer input images. Firstly, when extracting features from each image, we will consider feature fusion within each image to fuse multi-scale information, thus improving model robustness. Secondly, we will optimize the feature localization process of EFLM and the filter fusion mechanism of AFFM by introducing more advanced processing methods to improve localization accuracy and fusion efficiency. Finally, we will also explore efficient solutions from the perspective of addressing the challenges in recognition for more accurate pest identification.

## 5 Conclusion

To improve the performance of pest recognition in complex real-world scenarios, we proposed a multiimage fusion recognition method in this paper. In our method, CNN is used as the backbone network to extract features, EFLM effectively locates feature regions, AFFM adaptively filters and fuses features, and SV integrates multiple image recognition results to further improve recognition. To validate the effectiveness of our method, we conducted experiments not only on the low-quality pest dataset IP102 but also on the high-quality pest datasets D0 and ETP. The results demonstrate that our method can extract features to train high-performance networks and achieve much higher accuracy than the current state-of-the-art methods by inputting multiple images.

Moreover, we developed a web system to facilitate users with recognition needs to recognize pests in various crops and provide extreme recognition accuracy according to the amount of uploaded information, which meets the practical application requirements. In the future, we aim to more accurately locate targets and make better use of feature information to achieve higher accuracy with fewer images in practice.

## Data availability statement

The original contributions presented in the study are included in the article/supplementary material. Further inquiries can be directed to the corresponding author.

## Author contributions

YC: Conceptualization, Methodology, Software, Validation, Visualization, Writing – original draft, Writing – review & editing. MC: Investigation, Writing – review & editing. MG: Investigation, Writing – review & editing. JW: Project administration, Supervision, Writing – review & editing. NZ: Supervision, Writing – review & editing.

## References

[B1] Ahmad Loti N. N. Mohd Noor M. R. Chang S. W. (2021). Integrated analysis of machine learning and deep learning in chili pest and disease identification. J. Sci. Food Agric. 101, 3582–3594. doi: 10.1002/jsfa.10987 33275806

[B2] Cheng X. Zhang Y. Chen Y. Wu Y. Yue Y. (2017). Pest identification via deep residual learning in complex background. Comput. Electron. Agric. 141, 351–356. doi: 10.1016/j.compag.2017.08.005

[B3] Deng J. Dong W. Socher R. Li L. J. Kai L. Li F.-F. (2009). “Imagenet: A large-scale hierarchical image database,” in 2009 IEEE Conference on Computer Vision and Pattern Recognition. Miami, FL, USA: IEEE. 248–255. doi: 10.1109/CVPR.2009.5206848

[B4] Deng L. Wang Y. Han Z. Yu R. (2018). Research on insect pest image detection and recognition based on bio-inspired methods. Biosyst. Eng. 169, 139–148. doi: 10.1016/j.biosystemseng.2018.02.008

[B5] Dosovitskiy A. Beyer L. Kolesnikov A. Weissenborn D. Zhai X. Unterthiner T.. (2020). An image is worth 16x16 words: Transformers for image recognition at scale. doi: 10.48550/arXiv.2010.11929

[B6] Feng F. Dong H. Zhang Y. Zhang Y. Li B. (2022). Ms-aln: Multiscale attention learning network for pest recognition. IEEE Access 10, 40888–40898. doi: 10.1109/ACCESS.2022.3167397

[B7] He K. Zhang X. Ren S. Sun J. (2016). “Deep residual learning for image recognition,” in 2016 IEEE Conference on Computer Vision and Pattern Recognition. Las Vegas, NV, USA: IEEE. 770–778. doi: 10.1109/CVPR.2016.90

[B8] Hu J. Shen L. Sun G. (2018). “Squeeze-and-excitation networks,” in 2018 IEEE/CVF Conference on Computer Vision and Pattern Recognition. Salt Lake City, UT, USA: IEEE. 7132–7141. doi: 10.1109/CVPR.2018.00745

[B9] Hu K. Liu Y. Nie J. Zheng X. Zhang W. Liu Y.. (2023). Rice pest identification based on multi-scale double-branch gan-resnet. Front. Plant Sci. Salt Lake City, UT, USA: IEEE 14. doi: 10.3389/fpls.2023.1167121 PMC10140523 37123817

[B10] Huang M.-L. Chuang T.-C. Liao Y.-C. (2022). Application of transfer learning and image augmentation technology for tomato pest identification. Sustain. Computing: Inf. Syst. 33, 100646. doi: 10.1016/j.suscom.2021.100646

[B11] Huang G. Liu Z. Maaten L. V. D. Weinberger K. Q. (2017). “Densely connected convolutional networks,” in 2017 IEEE Conference on Computer Vision and Pattern Recognition. Honolulu, HI, USA: IEEE. 2261–2269. doi: 10.1109/CVPR.2017.243

[B12] Kong J. Wang H. Yang C. Jin X. Zuo M. Zhang X. (2022). A spatial feature-enhanced attention neural network with high-order pooling representation for application in pest and disease recognition. Agriculture 12, 500. doi: 10.3390/agriculture12040500

[B13] Li Y. Sun M. Qi Y. (2021). Common pests classification based on asymmetric convolution enhance depthwise separable neural network. J. Ambient Intell. Humanized Computing. 14, 8449-8457. doi: 10.1007/s12652-021-03611-0

[B14] Li Y. Wang H. Dang L. M. Sadeghi-Niaraki A. Moon H. (2020). Crop pest recognition in natural scenes using convolutional neural networks. Comput. Electron. Agric. 169, 105174. doi: 10.1016/j.compag.2019.105174

[B15] Liang K. Wang Y. Sun L. Xin D. Chang Z. (2022). “A lightweight-improved cnn based on vgg16 for identification and classification of rice diseases and pests,” in The International Conference on Image, Vision and Intelligent Systems. Changsha, China: Springer Nature Singapore. 195–207. doi: 10.1007/978-981-16-6963-7\18

[B16] Liu J. Wang X. (2021). Plant diseases and pests detection based on deep learning: a review. Plant Methods 17, 22. doi: 10.1186/s13007-021-00722-9 33627131 PMC7903739

[B17] Liu H. Zhan Y. Xia H. Mao Q. Tan Y. (2022). Self-supervised transformer-based pre-training method using latent semantic masking auto-encoder for pest and disease classification. Comput. Electron. Agric. 203, 107448. doi: 10.1016/j.compag.2022.107448

[B18] Mohsin M. R. B. Ramisa S. A. Saad M. Rabbani S. H. Tamkin S. Ashraf F. B.. (2022). “Classifying insect pests from image data using deep learning,” in 2022 15th International Congress on Image and Signal Processing, BioMedical Engineering and Informatics. Beijing, China: IEEE 1–6. doi: 10.1109/CISP-BMEI56279.2022.9979872

[B19] Nanni L. Manfè A. Maguolo G. Lumini A. Brahnam S. (2022). High performing ensemble of convolutional neural networks for insect pest image detection. Ecol. Inf. 67, 101515. doi: 10.1016/j.ecoinf.2021.101515

[B20] Selvaraju R. R. Cogswell M. Das A. Vedantam R. Parikh D. Batra D. (2017). “Grad-cam: Visual explanations from deep networks via gradient-based localization,” in 2017 IEEE International Conference on Computer Vision. Venica, Italy: IEEE. 618–626. doi: 10.1109/ICCV.2017.74

[B21] Sharma V. Tripathi A. K. Mittal H. (2022). “Technological advancements in automated crop pest and disease detection: A review & ongoing research,” in 2022 International Conference on Computing, Communication, Security and Intelligent Systems. Kochi, India: IEEE. 1–6. doi: 10.1109/IC3SIS54991.2022.9885605

[B22] Ullah N. Khan J. A. Alharbi L. A. Raza A. Khan W. Ahmad I. (2022). An efficient approach for crops pests recognition and classification based on novel deeppestnet deep learning model. IEEE Access 10, 73019–73032. doi: 10.1109/ACCESS.2022.3189676

[B23] Wang Q. Wang J. Deng H. Wu X. Wang Y. Hao G. (2023). Aa-trans: Core attention aggregating transformer with information entropy selector for fine-grained visual classification. Pattern Recognition 140, 109547. doi: 10.1016/j.patcog.2023.109547

[B24] Wei D. Chen J. Luo T. Long T. Wang H. (2022). Classification of crop pests based on multi-scale feature fusion. Comput. Electron. Agric. 194, 106736. doi: 10.1016/j.compag.2022

[B25] Wei X. S. Luo J. H. Wu J. Zhou Z. H. (2017). Selective convolutional descriptor aggregation for fine-grained image retrieval. IEEE Trans. Image Process. 26, 2868–2881. doi: 10.1109/TIP 28368819

[B26] Wu X. Zhan C. Lai Y.-K. Cheng M.-M. Yang J. (2019). “Ip102: A large-scale benchmark dataset for insect pest recognition,” in 2019 IEEE/CVF Conference on Computer Vision and Pattern Recognition. Long Beach, CA, USA: IEEE. 8779–8788. doi: 10.1109/CVPR.2019.00899

[B27] Xia W. Han D. Li D. Wu Z. Han B. Wang J. (2023). An ensemble learning integration of multiple cnn with improved vision transformer models for pest classification. Ann. Appl. Biol. 182, 144–158. doi: 10.1111/aab.12804

[B28] Xie C. Wang R. Zhang J. Chen P. Dong W. Li R.. (2018). Multi-level learning features for automatic classification of field crop pests. Comput. Electron. Agric. 152, 233–241. doi: 10.1016/j.compag.2018.07.014

[B29] Yang G. Chen G. Li C. Fu J. Guo Y. Liang H. (2021). Convolutional rebalancing network for the classification of large imbalanced rice pest and disease datasets in the field. Front. Plant Sci. 12. doi: 10.3389/fpls.2021.671134 PMC8287420 34290724

[B30] Zhang F. Li M. Zhai G. Liu Y. (2021). “Multi-branch and multi-scale attention learning for fine-grained visual categorization,” in MultiMedia Modeling: 27th International Conference, MMM 2021, Prague, Czech Republic, June 22–24, 2021, Proceedings, Part I. Prague, Czech Republic: Springer International Publishing. 136–147. doi: 10.1007/978-3-030-67832-6\12

[B31] Zheng T. Yang X. Lv J. Li M. Wang S. Li W. (2023). An efficient mobile model for insect image classification in the field pest management. Eng. Sci. Technology an Int. J. 39, 101335. doi: 10.1016/j.jestch.2023.101335

[B32] Zhu J. Wang Z. Chen J. Chen Y.-P. P. Jiang Y.-G. (2022). “Balanced contrastive learning for long-tailed visual recognition,” in 2022 IEEE/CVF Conference on Computer Vision and Pattern Recognition. New Orleans, LA, USA: IEEE. 6898–6907. doi: 10.1109/CVPR52688.2022.00678

